# Analytical study of robustness of a negative feedback oscillator by multiparameter sensitivity

**DOI:** 10.1186/1752-0509-8-S5-S1

**Published:** 2014-12-12

**Authors:** Kazuhiro Maeda, Hiroyuki Kurata

**Affiliations:** 1 Department of Bioscience and Bioinformatics, Kyushu Institute of Technology, 680-4 Kawazu, Iizuka, Fukuoka, 820-8502, Japan; 2 Biomedical Informatics R&D Center, Kyushu Institute of Technology, 680-4 Kawazu, Iizuka, Fukuoka, 820-8502, Japan

## Abstract

**Background:**

One of the distinctive features of biological oscillators such as circadian clocks and cell cycles is robustness which is the ability to resume reliable operation in the face of different types of perturbations. In the previous study, we proposed multiparameter sensitivity (MPS) as an intelligible measure for robustness to fluctuations in kinetic parameters. Analytical solutions directly connect the mechanisms and kinetic parameters to dynamic properties such as period, amplitude and their associated MPSs. Although negative feedback loops are known as common structures to biological oscillators, the analytical solutions have not been presented for a general model of negative feedback oscillators.

**Results:**

We present the analytical expressions for the period, amplitude and their associated MPSs for a general model of negative feedback oscillators. The analytical solutions are validated by comparing them with numerical solutions. The analytical solutions explicitly show how the dynamic properties depend on the kinetic parameters. The ratio of a threshold to the amplitude has a strong impact on the period MPS. As the ratio approaches to one, the MPS increases, indicating that the period becomes more sensitive to changes in kinetic parameters. We present the first mathematical proof that the distributed time-delay mechanism contributes to making the oscillation period robust to parameter fluctuations. The MPS decreases with an increase in the feedback loop length (i.e., the number of molecular species constituting the feedback loop).

**Conclusions:**

Since a general model of negative feedback oscillators was employed, the results shown in this paper are expected to be true for many of biological oscillators. This study strongly supports that the hypothesis that phosphorylations of clock proteins contribute to the robustness of circadian rhythms. The analytical solutions give synthetic biologists some clues to design gene oscillators with robust and desired period.

## Background

Robust oscillations are ubiquitous in biology such as circadian rhythms and cell cycles [1-4]. Robustness is the ability to resume reliable operation in the face of different types of perturbations: environmental and genetic changes, parameter uncertainty, and stochastic fluctuations [5-8]. It is critically important to understand the mechanisms by which biological oscillators robustly work in ever-fluctuating environments.

To quantify biochemical systems' robustness to fluctuations in all the kinetic parameters, multiparameter sensitivity (MPS) was used [8]. Although MPS is given by the sum of the squared single-parameter sensitivities, it represents how fragile the system's output is when small, random, and simultaneous fluctuations are provided to all kinetic parameters [8]. MPS is mathematically equal to the normalized variance calculated by the Monte Carlo method [9-11]. Use of MPS has revealed that negative feedback loops with multiple phosphorylations produce oscillators robust to parameter uncertainty [8]. The dual feedback model, a simplified version of the *Drosophila *PER-TIM feedback model [12], was found to be the most robust and entrainable among many feedback models with various connection logics [13].

Both numerical and analytical approaches are important for an understanding of design principles underlying robust biological oscillators. A number of numerical studies have been reported for the biological oscillators and many of mathematical models are available from databases such as JWS Online [14], BioModels [15,16] and BioFNet [17]. Numerical analyses have revealed the mechanisms of how a variety of feedback structures produce robust oscillators [8,13] and identified critical kinetic parameters that are related to changes in the period and amplitude [9,11]. Although numerical simulations are useful for a quantitative understanding of how complicated biological oscillators behave, they do not provide explicit information on how the period, amplitude and their robustness depend on kinetic parameters.

On the other hand, analytical solutions directly link the mechanisms and kinetic parameters to dynamic properties such as period and amplitude. However, analytical studies for biological oscillators are scarce compared to numerical counterparts. Most of analytical studies [18-20] focused on whether their models oscillate, but not on the robustness of period and amplitude. Kut et al [21] provided the analytical expressions for the period and amplitude for Elowitz-Leibler repressilator [22] and Barkai-Leibler circadian clock [23,24]. Their work is a great step toward an understanding of how the dynamic properties depend on kinetic parameters. However, the models they analyzed lack generality. Their analytical solutions are not applicable to other biological oscillators. To our knowledge, few analytical solutions for the period and amplitude have been reported. Analytical solutions for a general model of negative feedback loops, which are common structures to biological oscillators [25,26], greatly contribute to an understanding of design principles underlying robust biological oscillators.

In this paper, we present analytical solutions for the period and amplitude, and their associated MPSs for a general model of negative feedback oscillators. The analytical solutions are in agreement with numerical solutions of their ordinary differential equations. Our analytical or theoretical study of MPSs reveals the mechanisms by which negative feedback loops in biology generate robust oscillations. We present the first mathematical proof that long negative feedback loops make oscillators robust to parameter fluctuations by the distributed time-delay mechanism.

## Results and discussion

### Negative feedback oscillator model

**Figure 1:**
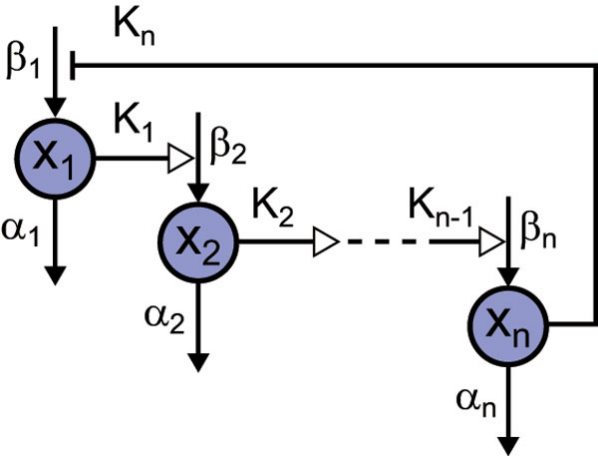
**Schematic diagram of the negative feedback oscillator model**. $x_{i}$ is the *i*th molecular species, $\alpha_{i}$ is the decay rate constant, $\beta_{i}$ is the production rate constant, and $K_{i}$ is the threshold for turning on/off the production of the target molecular species (i∈{1,2,…,n}). *n *is the number of molecular species. $x_{i}$ activates $x_{i+1}$ (i∈{1,2,…,n-1}). $x_{n}$ represses $x_{1}$. All molecular species are subject to degradation.

**Figure 2:**
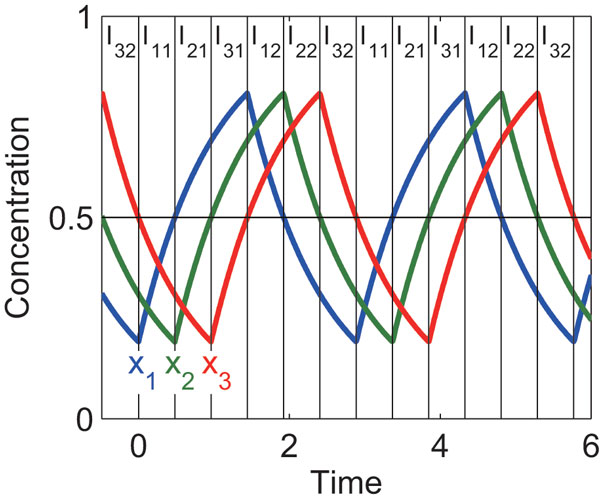
**Example of dynamics of the negative feedback oscillator model**. n=3, $\alpha_{i}=1$, $\beta_{i}=1$ and $K_{i}=0.5$ (i∈{1,2,3}). $I_{ij}$ are the intervals used to derive the analytical solutions for the period and amplitude (see **Text**).

We consider a general model of negative feedback oscillators with arbitrary loop length, where the feedback is imposed by the last species of the cascade on the first species. Figure 1 shows a schematic diagram of the negative feedback oscillator model. The dynamics is described by a set of differential equations:

(1) $$\begin{array}{c} \frac{dx_{1}}{dt}=\beta_{1}\cdot \theta \left(K_{n},x_{n}\right)-\alpha_{1}x_{1}\\ \frac{dx_{2}}{dt}=\beta_{2}\cdot \theta \left(x_{1},K_{1}\right)-\alpha_{2}x_{2}\\ \vdots \\ \frac{dx_{n}}{dt}=\beta_{n}\cdot \theta \left(x_{n-1},K_{n-1}\right)-\alpha_{n}x_{n} \end{array}$$

where $x_{i}$ is the concentration of the *i*th molecular species (e.g., transcription factor, specifically modified form of protein, metabolite, etc.), $\alpha_{i}$ is the decay rate constant, $\beta_{i}$ is the production rate constant, and $K_{i}$ is the threshold for turning on/off the production of the target molecular species (i∈{1,2,…,n}). $\alpha_{i}$, $\beta_{i}$ and $K_{i}$ take positive values. *n *is the number of molecular species or indicates feedback loop length.  θ is the unit step function given by

(2) $$\theta \left(a,b\right)=\left\{\begin{array}{c} \begin{array}{cc} 0 & \left(a<b\right) \end{array}\\ \begin{array}{cc} 1 & \left(a\geq b\right) \end{array} \end{array}\right)$$

where  a and  b are arbitrary positive values. Introducing the step function makes dynamic models tractable [27]. This on/off behavior arises from a sigmoidal Hill function. $K_{i}$ corresponds to the concentration of a regulator molecule at which the production rate of a target molecule reaches a half maximum in Hill function. $x_{i}$ takes a positive value in the range of (0, $\beta_{i}/\alpha_{i}$). The negative feedback oscillator model can produce oscillations when both n≥3 and $K_{i}<\beta_{i}/\alpha_{i}$ (i∈{1,…,n}) are satisfied. It has been proved that this type of negative feedback models cannot generate a sustained oscillation when n<3[20]. An example of the dynamics is shown in Figure 2.

Since at least one negative feedback loop is necessary for any biochemical networks to produce oscillations [25], the above model is found everywhere as a network motif in biological oscillatory networks. For example, circadian clock networks can be described by assuming that $x_{i}$ are clock genes or specifically modified forms of clock proteins. Our model can also be found in the field of synthetic biology. Elowitz-Leibler repressilator [22] and Atkinson's genetic circuit [28] can be considered as slightly modified versions of our model.

### Analytical solutions for period, amplitude and their associated MPSs

The period and amplitude were symbolically solved as follows. First, we divided a cycle of oscillations into time intervals ($I_{ij}$ in Figure 2). Next, we obtained the interval times, peaks and troughs. Finally, we connected all the intervals to calculate the period and amplitude (for details, see **Methods**). The period  and the *i*th species amplitude $\varepsilon_{i}$ for the oscillatory model of Eq (1) are given by

(3) $$\tau =-\overset{n}{\underset{i=1}{\sum }}\frac{\text{ln}\left(\gamma_{i}\delta_{i}\right)}{\alpha_{i}}$$

(4) $$\varepsilon_{i}=\left\{\begin{array}{c} C_{11}\left(\overset{n}{\underset{j=1}{\prod }}{\gamma_{j}}^{\alpha_{1}/\alpha_{j}}-1\right)\;\left(i=1\right)\\ C_{i1}\left(\overset{n}{\underset{j=i}{\prod }}{\gamma_{j}}^{\alpha_{i}/\alpha_{j}}\overset{i-1}{\underset{j=1}{\prod }}{\delta_{j}}^{\alpha_{i}/\alpha_{j}}-1\right)\;\left(i>1\right) \end{array}\right)$$

where $\gamma_{i}$ and $\delta_{i}$ are

(5) $$\gamma_{i}=\frac{K_{i}-\beta_{i}/\alpha_{i}}{C_{i1}}$$

(6) $$\delta_{i}=\frac{K_{i}}{C_{i2}}$$

$C_{ij}$ (i∈{1,…,n}, j∈{1,2}) are the integral constants:

(7) $$C_{i1}=\left\{\begin{array}{c} K_{1}\overset{n}{\underset{j=2}{\prod }}{\delta_{j}}^{\alpha_{1}/\alpha_{j}}-\frac{\beta_{1}}{\alpha_{1}}\;\left(i=1\right)\\ K_{i}\overset{i-1}{\underset{j=1}{\prod }}{\gamma_{j}}^{\alpha_{i}/\alpha_{j}}\overset{n}{\underset{j=i+1}{\prod }}{\delta_{j}}^{\alpha_{i}/\alpha_{j}}-\frac{\beta_{i}}{\alpha_{i}}\;\left(1<i<n\right)\\ K_{n}\overset{n-1}{\underset{j=1}{\prod }}{\gamma_{j}}^{\alpha_{n}/\alpha_{j}}-\frac{\beta_{n}}{\alpha_{n}}\;\left(i=n\right) \end{array}\right)$$

(8) $$C_{i2}=\left\{\begin{array}{c} C_{11}\overset{n}{\underset{j=1}{\prod }}{\gamma_{j}}^{\alpha_{1}/\alpha_{j}}+\frac{\beta_{1}}{\alpha_{1}}\;\left(i=1\right)\\ C_{i1}\overset{n}{\underset{j=i}{\prod }}{\gamma_{j}}^{\alpha_{i}/\alpha_{j}}\overset{i-1}{\underset{j=1}{\prod }}{\delta_{j}}^{\alpha_{i}/\alpha_{j}}+\frac{\beta_{i}}{\alpha_{i}}\;\left(i>1\right) \end{array}\right)$$

Although $C_{ij}$ are hard to solve in symbolic form, they can be determined by numerically solving the system of Eqs (7)-(8).

Assuming that the peak and trough of $x_{i}$ are $\beta_{i}/\alpha_{i}$ and zero, respectively, the integral constants can be determined without any numerical computations, and the analytical solutions for the period  and the *i*th species amplitude $\varepsilon_{i}$ are given by (for details, see **Methods**)

(9) $$\tau =-\overset{n}{\underset{i=1}{\sum }}\frac{\text{ln}\left[\rho_{i}\left(1-\rho_{i}\right)\right]}{\alpha_{i}}$$

(10) $$\varepsilon_{i}=\frac{\beta_{i}}{\alpha_{i}}$$

where $\rho_{i}$ is the ratio of the threshold $K_{i}$ to the amplitude $\beta_{i}/\alpha_{i}$:

(11) $$\rho_{i}=\frac{K_{i}\alpha_{i}}{\beta_{i}}$$

Multiparameter sensitivity (MPS) represents how fragile system's properties are to fluctuations in kinetic parameters (for details, see **Methods**). The period MPS ${\text{Φ}}_{\tau }$ and amplitude MPS ${\text{Φ}}_{\varepsilon_{i}}$ are given by

(12) $${\text{Φ}}_{\tau }=\frac{\overset{n}{\underset{i=1}{\sum }}\left[{\left(\frac{\text{ln}\left[\rho_{i}\left(1-\rho_{i}\right)\right]}{\alpha_{i}}-\frac{1-2\rho_{i}}{\alpha_{i}\left(1-\rho_{i}\right)}\right)}^{2}+2{\left(\frac{1-2\rho_{i}}{\alpha_{i}\left(1-\rho_{i}\right)}\right)}^{2}\right]}{{\left[\overset{n}{\underset{i=1}{\sum }}\frac{\text{ln}\left[\rho_{i}\left(1-\rho_{i}\right)\right]}{\alpha_{i}}\right]}^{2}}$$

(13) $${\text{Φ}}_{\varepsilon_{i}}=2$$

### Validation of the analytical solutions

**Figure 3:**
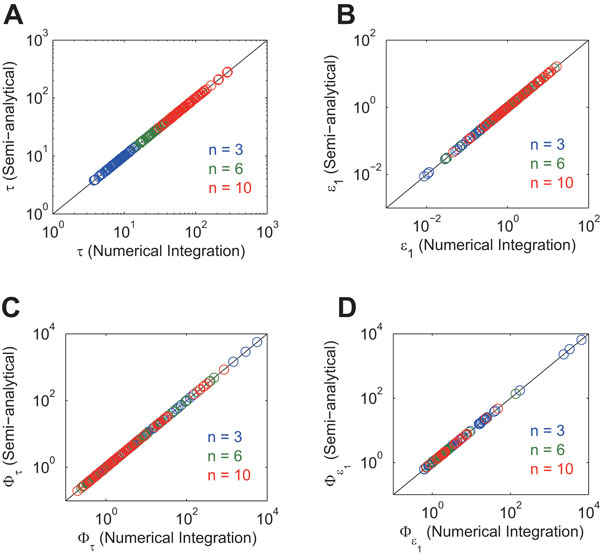
**Comparison of the semi-analytical and numerical integration solutions **. A: period, B: amplitude, C: period MPS, D: amplitude MPS. The values of $\alpha_{i}$ and $\beta_{i}$ (i∈{1,2,…,n}) were randomized with a range of (0, 1), while the value of $K_{i}$ (i∈{1,2,…,n}) was randomized with a range of (0, $\beta_{i}/\alpha_{i}$).

First, we like to validate the analytical solutions given by Eqs (3)-(4) (which are free of the assumptions of $\beta_{i}/\alpha_{i}$ peak and zero trough). In order to determine $C_{ij}$, fsolve function of MATLAB (The MathWorks, Inc.) was used. The MPSs for period and amplitude were numerically calculated by providing a small perturbation to the values of $\alpha_{i}$, $\beta_{i}$ and $K_{i}$ (see **Methods**). The computed period, amplitude and MPSs are referred to as the semi-analytical solutions because numerical methods were partially used. The semi-analytical solutions were compared with the numerical integration solutions. The numerical integration solutions for the period and amplitude were obtained by numerically integrating Eq (1). Table 1 summarizes the methods to obtain the semi-analytical and numerical integration solutions. We assigned uniform random values over (0, 1) to $\alpha_{i}$ and $\beta_{i}$, and those over (0, $\beta_{i}/\alpha_{i}$) to $K_{i}$. The semi-analytical solutions were consistent with the numerical integration solutions (Figure 3). Thus, the analytical solutions given by Eqs (3)-(4) were validated.

**Table 1:** Summary of how to obtain numerical integration, semi-analytical, and full analytical solutions

	Numerical integration solution	Semi-analytical solution	Full analytical Solution
τ	Numerical integration of Eq (1)	Eq (3) (Eqs (7)-(8) are numerically solved)	Eq (9)
$\varepsilon_{i}$	Numerical integration of Eq (1)	Eq (4) (Eqs (7)-(8) are numerically solved)	Eq (10)
${\text{Φ}}_{\tau }$	Numerical computation (see **Methods**)	Numerical computation (see **Methods**)	Eq (12)
${\text{Φ}}_{\varepsilon_{i}}$	Numerical computation (see **Methods**)	Numerical computation (see **Methods**)	Eq (13)

In addition to the correctness of the solutions, the semi-analytical approach is computationally more efficient than the numerical integration approach. When n=3, the semi-analytical approach (fsolve function of MATLAB) required 0.1 sec per parameter set in order to calculate the period, amplitude and their associated MPSs. On the other hand, the numerical integration approach (ode15s function of MATLAB) required 12 sec per parameter set. Although it looks computationally inexpensive to numerically integrate the differential equations of Eq (1), it takes much computational cost because of 'stiffness' that comes from the step function of Eq (2).

**Figure 4:**
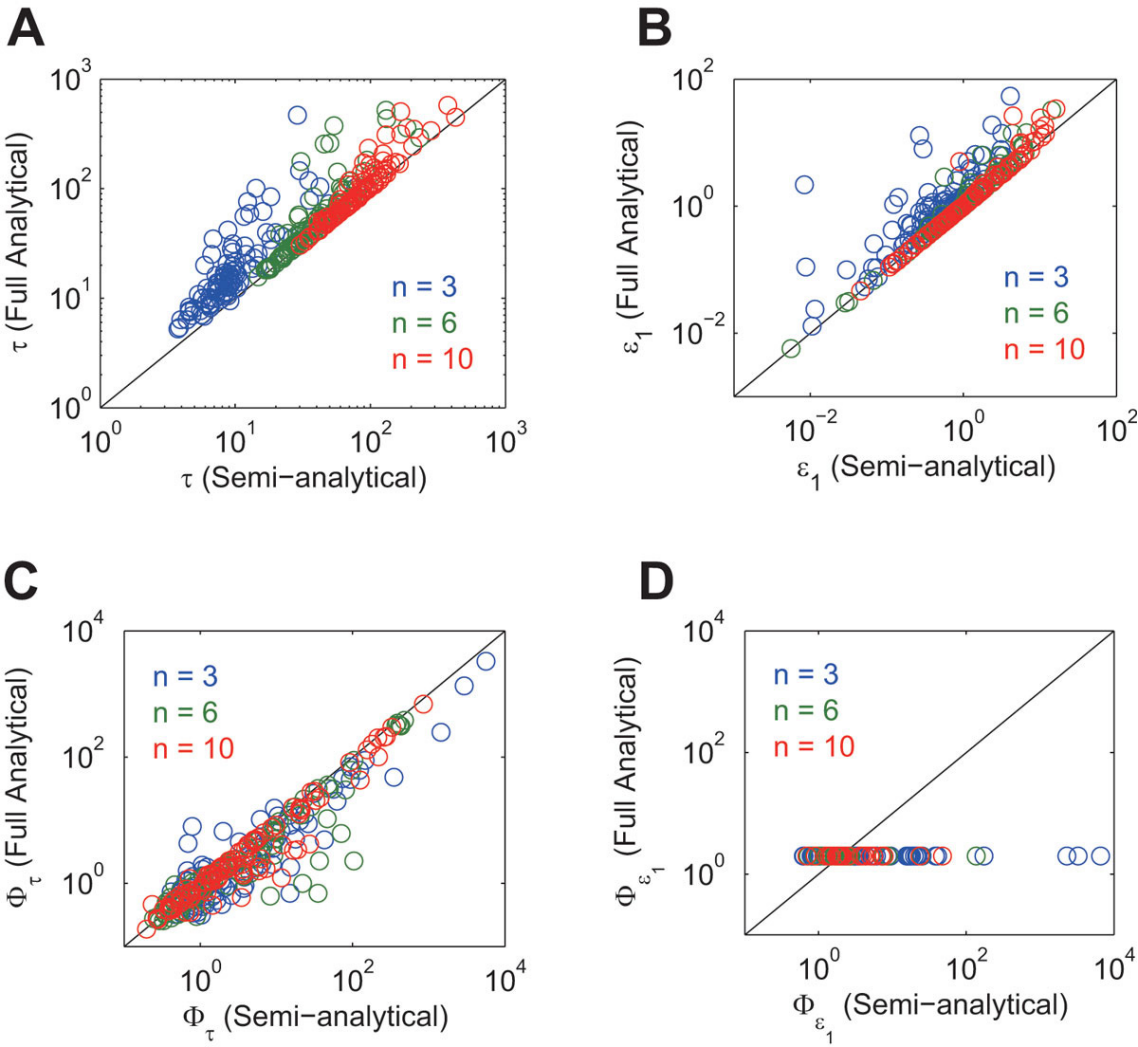
**Comparison of the full analytical and semi-analytical solutions**. A: period, B: amplitude, C: period MPS, D: amplitude MPS. The values of $\alpha_{i}$ and $\beta_{i}$ (i∈{1,2,…,n}) were randomized with a range of (0, 1), while the value of $K_{i}$ (i∈{1,2,…,n}) was randomized with a range of (0, $\beta_{i}/\alpha_{i}$).

**Figure 5:**
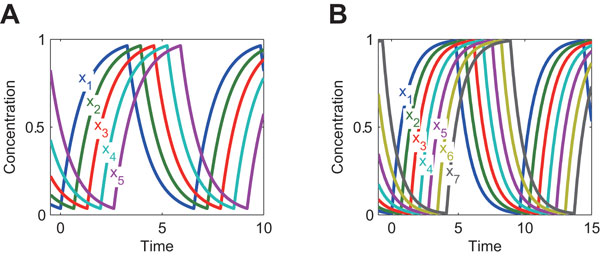
**Examples of dynamics of the negative feedback oscillator model**. A: n=5, B: n=7. $\alpha_{i}=1$, $\beta_{i}=1$ and $K_{i}=0.5$ (i∈{1,2,…,n}).

Next, we like to validate the analytical solutions given by Eqs (9)-(10) and Eqs (12)-(13) (which were derived by assuming $\beta_{i}/\alpha_{i}$ peak and zero trough). The solutions given by Eqs (9)-(10) and Eqs (12)-(13) are referred to as the full analytical solutions because they are free of any numerical methods (Table 1). We compared the full and semi-analytical solutions. We assigned random values to kinetic parameters as we did for the comparison between the semi-analytical and numerical integration solutions. The results are shown in Figure 4. The full and semi-analytical solutions are consistent especially when the feedback loop is long, because the assumptions (the peak is $\beta_{i}/\alpha_{i}$ and the trough is zero) are met when the feedback loop is long (Figure 5, and compare it with Figure 2). Even for the short loop (n=3), the differences between the full and semi-analytical solutions are less than an order of magnitude for most parameter sets. Note that, in Figure 4D, the full analytical solutions for the amplitude MPS are always two, independent of kinetic parameter values (Eq (13)). Although some symbols seem scattered away from the diagonal line in Figure 4D, most of the symbols are so concentrated on the diagonal line that the full and semi-analytical solutions are consistent. In summary, the analytical solutions given by Eqs (9)-(10) and Eqs (12)-(13) were validated.

### Effect of changes in kinetic parameters on period and its MPS

Having validated the analytical solutions given by Eqs (9)-(10) and Eqs (12)-(13), we investigated how the period and period MPS depend on the values of kinetic parameters (we do not investigate the amplitude and amplitude MPS because how they depend on kinetic parameters is clear from Eqs (10) and (13)). **Figure S1 **shows that how the period depends on kinetic parameters. For simplicity, we assumed n=3, $\alpha_{i}=\alpha$, $\beta_{i}=\beta$, $K_{i}=K$ (i∈{1,2,3}), and ρ=Kα/β. When the period is given as a function of , it reaches the minimal values at ρ=0.7228 (**Figure S1AB **in Additional file 1). When the period is given as a function of β or *K*, it reaches the minimal values at  (**Figure S1CDEF**). Note that the negative feedback oscillator model produces oscillations only when K<β/α (ρ<1). **Figure S2 **(Additional file 1) shows how the period MPS depends on kinetic parameters. The period MPS always reaches the minimal value 0.2222 at ρ=0.5959, implying that the period MPS depends solely on ρ (when *n *is fixed).

**Figure 6:**
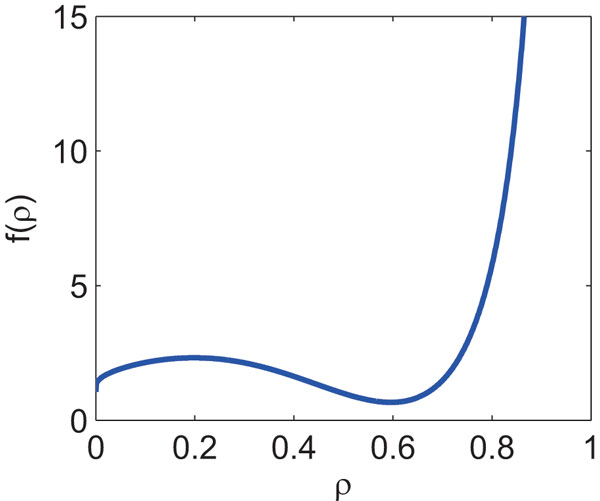
**f(ρ) vs. ρ. f(ρ) is given by Eq (15) in Text**.

Assuming $\alpha_{i}=\alpha$ and $\rho_{i}=\rho$ (i∈{1,2,…,n}), Eq (12) reduces to

(14) $${\text{Φ}}_{\tau }=\frac{f\left(\rho \right)}{n}$$

where

(15) $$f\left(\rho \right)=\frac{{\left(\text{ln}\left[\rho \left(1-\rho \right)\right]-\frac{1-2\rho }{1-\rho }\right)}^{2}+2{\left(\frac{1-2\rho }{1-\rho }\right)}^{2}}{{\left(\text{ln}\left[\rho \left(1-\rho \right)\right]\right)}^{2}}$$

f(ρ) reaches the minimal value 0.6667 at ρ=0.5959, and $\underset{\rho \to 1}{\text{lim}}f\left(\rho \right)=\infty$ (Figure 6). Eq (14) shows that ρ has a significant effect on the period MPS. In order to reduce the period MPS, i.e., to make the period robust to parameter fluctuations, ρ should not be close to one. Eq (14) also shows the feedback loop length n is important. The following section explains how the period MPS depends on the feedback loop length.

### Effect of changes in feedback loop length on period and its MPS

Assuming $\rho_{i}=0.5$ ($K_{i}=\beta_{i}/2\alpha_{i}$), Eq (9) and Eq (12) reduce to

(16) $$\tau =\text{ln}4\cdot \overset{n}{\underset{i=1}{\sum }}{\alpha_{i}}^{-1}$$

(17) $${\text{Φ}}_{\tau }=\frac{\overset{n}{\underset{i=1}{\sum }}{\alpha_{i}}^{-2}}{{\left(\overset{n}{\underset{i=1}{\sum }}{\alpha_{i}}^{-1}\right)}^{2}}$$

respectively. Therefore, the value of period MPS is constrained by

(18) $$\frac{1}{n}\leq {\text{Φ}}_{\tau }\leq 1$$

The minimal value of the period MPS decreases as *n *increases. Here, let $\Psi_{\tau }$be the sum of all the single-parameter sensitivities of the period. Then, we get an interesting relationship among the single-parameter sensitivities (see Eqs (61)-(63) in **Methods**):

(19) $$\begin{array}{cc} \Psi_{\tau } & =\overset{n}{\underset{i=1}{\sum }}\left(S_{\alpha_{i}}^{\tau }+S_{\beta_{i}}^{\tau }+S_{K_{i}}^{\tau }\right)\\ & =\overset{n}{\underset{i=1}{\sum }}S_{\alpha_{i}}^{\tau }\\ & =-1 \end{array}$$

Note that $S_{\beta_{i}}^{\tau }=S_{K_{i}}^{\tau }=0$ and $S_{\alpha_{i}}^{\tau }<0$ when $\rho_{i}=0.5$. Eq (19) indicates that all the degradation reactions share the influence on the period. The MPS given by Eq (17) is minimized when all the degradation reactions equally share the influence ($S_{\alpha_{i}}^{\tau }=-1/n$), which is achieved by equating all $\alpha_{i}$. Assuming $\alpha_{i}=\alpha$, Eq (16) and Eq (17) further reduce to

(20) $$\tau =\frac{n\text{ln}4}{\alpha }$$

(21) $${\text{Φ}}_{\tau }=\frac{1}{n}$$

respectively. Eq (20) indicates that the period increases with an increase in the feedback loop length n and/or with a decrease in the decay rate constant α. Eq (21) indicates that changes in α do not alter the MPS. On the other hand, the MPS decreases as *n *increases.

**Figure 7:**
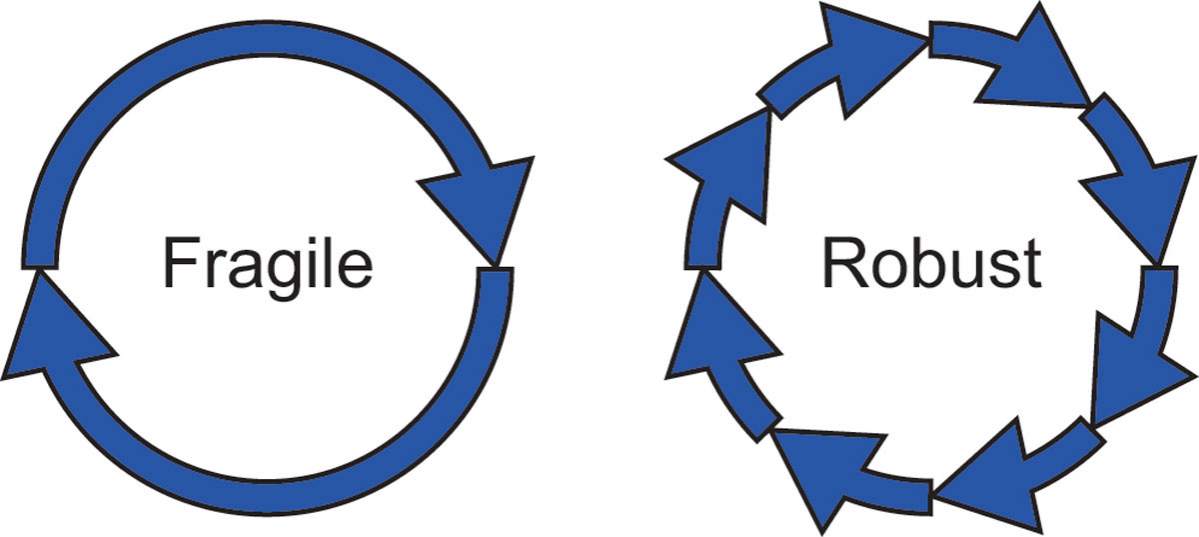
**Distributed time-delay mechanism**. Oscillators with two (left) and eight (right) time delays. The right is more robust than the left because of the distributed time-delay mechanism (see **Text**).

In the negative feedback oscillators, the period is given by the sum of the time delays generated by the reactions belonging to the feedback loop (Figure 7). As the number of the reactions increases (thus, *n *increases), the time delays can be distributed to more reactions. This 'distributed-time delay' mechanism provides the oscillation period with robustness to parameter fluctuations, decreasing the MPS.

In the previous paper [8], we proved that the merge reactions with addition logic contribute to keeping a steady-state component concentration constant against fluctuations in kinetic parameters. The concentration MPS decreases as the number of merging influx reactions increases (see Appendix A4 in [8]). The period MPS given by Eq (17) resembles the concentration MPS in the merge reactions. Although the oscillation period and steady-state concentration are different properties, our studies suggest that the underlying mechanisms that provide robustness are common to both the cases. The oscillation period is given by the sum of time delays, and the steady-state concentration is determined by the sum of influxes.

## Conclusions

We presented the analytical solutions of period, amplitude, and their associated MPSs for a general model of negative feedback oscillators. We validated the analytical solutions by comparing them with numerical solutions. Next, using the analytical solutions, we investigated how changes in kinetic parameters affect the period and its associated MPS. $\rho_{i}$, the ratio of threshold value to the amplitude, was found to be an important determinant of the period MPS. When $\rho_{i}$ is close to one ($K_{i}\approx \beta_{i}/\alpha_{i}$ ), MPS is very large, indicating that the period is very sensitive to fluctuations in kinetic parameters. Finally, we gave the first mathematical proof that long negative feedback loops make oscillations robust to parameter fluctuations by the distributed time-delay mechanism. Since the analytical solutions were derived for a general model of negative feedback loops that are common structures in biological oscillators, the results presented in this paper are expected to be true for many of biological oscillators.

In circadian rhythm networks, clock proteins have multiple phosphorylation sites. It has been reported that the phosphorylation is responsible for sleep disorders [29-31] and temperature compensation [32]. In the previous study, we carried out numerical simulations to predict that the multiple phosphorylations contribute to enhanced robustness of circadian rhythms [8]. The theoretical study in this paper strongly supports our previous result. The multiple phosphorylations form a long negative feedback loop, providing the distributed time-delay mechanism. It is reasonable for organisms to devise the multiple phosphorylation sites in order to make circadian cycles robust.

To date synthetic biologists have focused on whether their artificial gene regulatory circuits oscillate [22,28,33-35]. Their next challenge would be to design oscillator circuits with desired and accurate period. Our study demonstrates key factors for designing such a high-performance clock. For instance, $\rho_{i}$ ($=K_{i}\alpha_{i}/\beta_{i}$) should not be close to one to make the period insensitive to parameter fluctuations (Eqs (14)-(15)). When $\rho_{i}=0.5959$, the period becomes robust to parameter fluctuations. The robustness can be further increased by lengthening a feedback loop. To design an oscillator with long period, one can employ a long negative feedback loop (large *n*) and/or slow decay rates (small $\alpha_{i}$) as suggested by Eq (9). Our analytical study recommends increasing the feedback loop length instead of decreasing decay rates, achieving a robust oscillator (Eqs (17)-(18) and Eq (21)). In summary, the analytical solutions presented in this paper greatly contribute to designing robust gene oscillators with desired period.

## Methods

### Derivation of analytical solutions for period and amplitude

In this section, we explain how to derive the analytical solutions for the period and amplitude. For simplicity, we assume n=3 and Eq (1) becomes

(22) $$\begin{array}{c} \frac{dx_{1}}{dt}=\beta_{1}\cdot \theta \left(K_{3},x_{3}\right)-\alpha_{1}x_{1}\\ \frac{dx_{2}}{dt}=\beta_{2}\cdot \theta \left(x_{1},K_{1}\right)-\alpha_{2}x_{2}\\ \frac{dx_{3}}{dt}=\beta_{3}\cdot \theta \left(x_{2},K_{2}\right)-\alpha_{3}x_{3} \end{array}$$

An example of the dynamics is shown in Figure 2. First, we divide a cycle of oscillations into time intervals ($I_{ij}$ in Figure 2). In $I_{i1}$, $x_{i}$ increases from its trough to $K_{i}$. In $I_{i2}$, $x_{i}$ decreases from its peak to $K_{i}$. The time taken for $I_{ij}$is denoted by $\tau_{ij}$. Next, we derive analytical expressions for each time interval, and the peaks and troughs of oscillation. Finally, we connect all the time intervals to obtain period. The amplitude is obtained by subtracting the trough from the peak. We set t=0 to the starting time of $I_{11}$.

In $I_{11}$, $I_{21}$ and $I_{31}$, the first molecular species is produced (θ=1) and thus the differential equation for $x_{1}$ in Eqs (22) becomes

(23) $$\frac{dx_{1}}{dt}=\beta_{1}-\alpha_{1}x_{1}$$

Integrating Eq (23), we obtain

(24) $$x_{1}\left(t\right)=\frac{\beta_{1}}{\alpha_{1}}+C_{11}e^{-\alpha_{1}t}$$

Similarly, in $I_{21}$, $I_{31}$ and $I_{12}$, $x_{2}$ follows

(25) $$x_{2}\left(t\right)=\frac{\beta_{2}}{\alpha_{2}}+C_{21}e^{-\alpha_{2}\left(t-\tau_{11}\right)}$$

Since t=0 is the starting time of $I_{11}$, the subtraction of $\tau_{11}$ appears in Eq (25). The same shall apply to Eq (26) and Eqs (28)-(30). In $I_{31}$, $I_{12}$ and $I_{22}$, $x_{3}$ follows

(26) $$x_{3}\left(t\right)=\frac{\beta_{3}}{\alpha_{3}}+C_{31}e^{-\alpha_{3}\left(t-\tau_{11}-\tau_{21}\right)}$$

In $I_{12}$, $I_{22}$ and $I_{32}$, the first molecular species is not produced (θ=0) and thus the differential equation for $x_{1}$ in Eqs (22) becomes

(27) $$\frac{dx_{1}}{dt}=-\alpha_{1}x_{1}$$

Integrating Eq (27), we obtain

(28) $$x_{1}\left(t\right)=C_{12}e^{-\alpha_{1}\left(t-\tau_{11}-\tau_{21}-\tau_{31}\right)}$$

Similarly, in $I_{22}$, $I_{32}$ and $I_{11}$, $x_{2}$ follows

(29) $$x_{2}\left(t\right)=C_{22}e^{-\alpha_{2}\left(t-\tau_{11}-\tau_{21}-\tau_{31}-\tau_{12}\right)}$$

In $I_{32}$, $I_{11}$ and $I_{21}$, $x_{3}$ follows

(30) $$x_{3}\left(t\right)=C_{32}e^{-\alpha_{3}\left(t-\tau_{11}-\tau_{21}-\tau_{31}-\tau_{12}-\tau_{22}\right)}$$

$C_{ij}$(i∈{1,2,3}, j∈{1,2}) are the integral constants. Here, we would like to calculate $\tau_{ij}$. Since $x_{i}$ increases and reaches to $K_{i}$ at $t=\overset{i}{\underset{j=1}{\sum }}\tau_{j1}$ (Figure 2), we obtain

(31) $$x_{i}\left(\overset{i}{\underset{j=1}{\sum }}\tau_{j1}\right)=K_{i}$$

By inserting Eqs (24)-(26), Eq (31) becomes

(32) $$\frac{\beta_{i}}{\alpha_{i}}+C_{i1}e^{-\alpha_{i}\tau_{i1}}=K_{i}$$

By solving Eq (32) for $\tau_{i1}$, we obtain

(33) $$\tau_{i1}=-\frac{\text{ln}\gamma_{i}}{\alpha_{i}}$$

where

(34) $$\gamma_{i}=\frac{K_{i}-\beta_{i}/\alpha_{i}}{C_{i1}}$$

Since $x_{i}$ decreases and reaches to $K_{i}$ at $t=\overset{3}{\underset{j=1}{\sum }}\tau_{j1}+\overset{i}{\underset{j=1}{\sum }}\tau_{j2}$ (Figure 2), we obtain

(35) $$x_{i}\left(\overset{3}{\underset{j=1}{\sum }}\tau_{j1}+\overset{i}{\underset{j=1}{\sum }}\tau_{j2}\right)=K_{i}$$

By inserting Eqs (28)-(30), Eq (35) becomes

(36) $$C_{i2}e^{-\alpha_{i}\tau_{i2}}=K_{i}$$

By solving Eq (36) for $\tau_{i2}$, we obtain

(37) $$\tau_{i2}=-\frac{\text{ln}\delta_{i}}{\alpha_{i}}$$

where

(38) $$\delta_{i}=\frac{K_{i}}{C_{i2}}$$

By inserting Eqs (33) and (37) into Eqs (24)-(26), we get the peaks of $x_{1}$, $x_{2}$ and $x_{3}$:

(39) $$x_{1}\left(\overset{3}{\underset{i=1}{\sum }}\tau_{i1}\right)=\frac{\beta_{1}}{\alpha_{1}}+C_{11}\gamma_{1}{\gamma_{2}}^{\alpha_{1}/\alpha_{2}}{\gamma_{3}}^{\alpha_{1}/\alpha_{3}}$$

(40) $$x_{2}\left(\overset{3}{\underset{i=1}{\sum }}\tau_{i1}+\tau_{12}\right)=\frac{\beta_{2}}{\alpha_{2}}+C_{21}\gamma_{2}{\gamma_{3}}^{\alpha_{2}/\alpha_{3}}{\delta_{1}}^{\alpha_{2}/\alpha_{1}}$$

(41) $$x_{3}\left(\overset{3}{\underset{i=1}{\sum }}\tau_{i1}+\tau_{12}+\tau_{22}\right)=\frac{\beta_{3}}{\alpha_{3}}+C_{31}\gamma_{3}{\delta_{1}}^{\alpha_{3}/\alpha_{1}}{\delta_{2}}^{\alpha_{3}/\alpha_{2}}$$

By inserting Eqs (33) and (37) into Eqs (28)-(30), we get the troughs of $x_{1}$, $x_{2}$ and $x_{3}$:

(42) $$x_{1}\left(\overset{3}{\underset{i=1}{\sum }}\tau_{i1}+\overset{3}{\underset{i=1}{\sum }}\tau_{i2}\right)=K_{1}{\delta_{2}}^{\alpha_{1}/\alpha_{2}}{\delta_{3}}^{\alpha_{1}/\alpha_{3}}$$

(43) $$x_{2}\left(\overset{3}{\underset{i=1}{\sum }}\tau_{i1}+\overset{3}{\underset{i=1}{\sum }}\tau_{i2}+\tau_{11}\right)=K_{2}{\delta_{3}}^{\alpha_{2}/\alpha_{3}}{\gamma_{1}}^{\alpha_{2}/\alpha_{1}}$$

(44) $$x_{3}\left(\overset{3}{\underset{i=1}{\sum }}\tau_{i1}+\overset{3}{\underset{i=1}{\sum }}\tau_{i2}+\tau_{11}+\tau_{21}\right)=K_{3}{\gamma_{1}}^{\alpha_{3}/\alpha_{1}}{\gamma_{2}}^{\alpha_{3}/\alpha_{2}}$$

By summing up $\tau_{ij}$, we obtain the period:

(45) $$\begin{array}{c} \tau =\overset{3}{\underset{i=1}{\sum }}\left(\tau_{i1}+\tau_{i2}\right)\\ =-\overset{3}{\underset{i=1}{\sum }}\frac{\text{ln}\left(\gamma_{i}\delta_{i}\right)}{\alpha_{i}} \end{array}$$

The oscillation amplitude is calculated by subtracting the trough from the peak. From Eqs (24) and (39), the amplitude of $x_{1}$ is

(46) $$\begin{array}{c} \varepsilon_{1}=x_{1}\left(\overset{3}{\underset{i=1}{\sum }}\tau_{i1}\right)-x_{1}\left(0\right)\\ =C_{11}\left(\gamma_{1}{\gamma_{2}}^{\alpha_{1}/\alpha_{2}}{\gamma_{3}}^{\alpha_{1}/\alpha_{3}}-1\right) \end{array}$$

Similarly, from Eqs (25) and (40), the amplitude of $x_{2}$ is

(47) $$\begin{array}{c} \varepsilon_{2}=x_{2}\left(\overset{3}{\underset{i=1}{\sum }}\tau_{i1}+\tau_{12}\right)-x_{2}\left(\tau_{11}\right)\\ =C_{21}\left(\gamma_{2}{\gamma_{3}}^{\alpha_{2}/\alpha_{3}}{\delta_{1}}^{\alpha_{2}/\alpha_{1}}-1\right) \end{array}$$

From Eqs (26) and (41), the amplitude of $x_{3}$ is

(48) $$\begin{array}{c} \varepsilon_{3}=x_{3}\left(\overset{3}{\underset{i=1}{\sum }}\tau_{i1}+\tau_{12}+\tau_{22}\right)-x_{3}\left(\tau_{11}+\tau_{21}\right)\\ =C_{31}\left(\gamma_{3}{\delta_{1}}^{\alpha_{3}/\alpha_{1}}{\delta_{2}}^{\alpha_{3}/\alpha_{2}}-1\right) \end{array}$$

The general expressions for the period and amplitude are shown as Eqs (3) and (4), respectively. To calculate τ and $\varepsilon_{i}$ (i∈{1,2,3}), we have to determine $C_{ij}$ (i∈{1,2,3},j∈{1,2}). $C_{ij}$ are determined by solving the following six equations. $x_{1}$ at the end of $I_{32}$ equals to that at the starting point of $I_{11}$. By equating Eq (42) to $x_{1}\left(0\right)$ of Eq (24),

(49) $$C_{11}=K_{1}{\delta_{2}}^{\alpha_{1}/\alpha_{2}}{\delta_{3}}^{\alpha_{1}/\alpha_{3}}-\frac{\beta_{1}}{\alpha_{1}}$$

$x_{2}$ at the end of $I_{11}$ equals to that at the starting point of $I_{21}$. By equating Eq (43) to $x_{2}\left(\tau_{11}\right)$ of Eq (25),

(50) $$C_{21}=K_{2}{\delta_{3}}^{\alpha_{2}/\alpha_{3}}{\gamma_{1}}^{\alpha_{2}/\alpha_{1}}-\frac{\beta_{2}}{\alpha_{2}}$$

$x_{3}$ at the end of $I_{21}$ equals to that at the starting point of $I_{31}$. By equating Eq (44) to $x_{3}\left(\tau_{11}+\tau_{21}\right)$ of Eq (26),

(51) $$C_{31}=K_{3}{\gamma_{1}}^{\alpha_{3}/\alpha_{1}}{\gamma_{2}}^{\alpha_{3}/\alpha_{2}}-\frac{\beta_{3}}{\alpha_{3}}$$

$x_{1}$ at the end of $I_{31}$ equals to that at the starting point of $I_{12}$. By equating Eq (39) to $x_{1}\left(\overset{3}{\underset{i=1}{\sum }}\tau_{i1}\right)$ of Eq (28),

(52) $$C_{12}=C_{11}\gamma_{1}{\gamma_{2}}^{\alpha_{1}/\alpha_{2}}{\gamma_{3}}^{\alpha_{1}/\alpha_{3}}+\frac{\beta_{1}}{\alpha_{1}}$$

$x_{2}$ at the end of $I_{12}$ equals to that at the starting point of $I_{22}$ . By equating Eq (40) to $x_{2}\left(\overset{3}{\underset{i=1}{\sum }}\tau_{i1}+\tau_{12}\right)$ of Eq (29),

(53) $$C_{22}=C_{21}\gamma_{2}{\gamma_{3}}^{\alpha_{2}/\alpha_{3}}{\delta_{1}}^{\alpha_{2}/\alpha_{1}}+\frac{\beta_{2}}{\alpha_{2}}$$

$x_{3}$ at the end of $I_{22}$ equals to that at the starting point of $I_{32}$. By equating Eq (41) to $x_{3}\left(\overset{3}{\underset{i=1}{\sum }}\tau_{i1}+\tau_{12}+\tau_{22}\right)$ of Eq (30),

(54) $$C_{32}=C_{31}\gamma_{3}{\delta_{1}}^{\alpha_{3}/\alpha_{1}}{\delta_{2}}^{\alpha_{3}/\alpha_{2}}+\frac{\beta_{3}}{\alpha_{3}}$$

The general forms of Eqs (49)-(51) and Eqs (52)-(54) are expressed as Eq (7) and Eq (8), respectively. Since there are six integral constants ($C_{ij}$) and six equations (Eqs (49)-(54)), the integral constants can be calculated. For this purpose, we used fsolve of MATLAB in this study.

### Approximation of analytical solutions for period and amplitude

Assuming θ=1 for Eqs (1), $x_{i}$ moves toward its steady-state value $\beta_{i}/\alpha_{i}$, which is given by setting $dx_{i}/dt=0$. Similarly, assuming θ=0, $x_{i}$ moves toward zero. As shown in Figure 2 and 5, the peak and trough of $x_{i}$ are close to $\beta_{i}/\alpha_{i}$ and zero, respectively. Therefore, we assume that the peak and trough are $\beta_{i}/\alpha_{i}$ and zero, respectively, i.e., $x_{i}$ increases (decreases) to reach its steady state before it begins to decreases (increases). This assumption allows us to symbolically derive the period and amplitude as shown below. The assumption is validated in "Results and discussion."

The assumption that the trough is zero gives

(55) $$\begin{array}{c} x_{1}\left(0\right)=0\\ x_{i}\left(\overset{i-1}{\underset{j=1}{\sum }}\tau_{j1}\right)=0\;\left(i>1\right) \end{array}$$

Using Eq (55) and Eqs (24)-(26), the integral constants $C_{i1}$ are

(56) $$C_{i1}=-\frac{\beta_{i}}{\alpha_{i}}$$

The assumption that the peak is $\beta_{i}/\alpha_{i}$ gives

(57) $$\begin{array}{c} x_{1}\left(\overset{n}{\underset{j=1}{\sum }}\tau_{j1}\right)=\frac{\beta_{i}}{\alpha_{i}}\\ x_{i}\left(\overset{n}{\underset{j=1}{\sum }}\tau_{j1}+\overset{i-1}{\underset{j=1}{\sum }}\tau_{j2}\right)=\frac{\beta_{i}}{\alpha_{i}}\;\left(i>1\right) \end{array}$$

Using Eqs (57) and Eqs (28)-(30), the integral constants $C_{i2}$ are

(58) $$C_{i2}=\frac{\beta_{i}}{\alpha_{i}}$$

By inserting Eqs (56) and (58) into Eqs (34) and (38), respectively, the period τ described by Eq (3) (or Eq (45)) becomes

(59) $$\tau =-\overset{n}{\underset{i=1}{\sum }}\frac{\text{ln}\left[\rho_{i}\left(1-\rho_{i}\right)\right]}{\alpha_{i}}$$

where $\rho_{i}=K_{i}\alpha_{i}/\beta_{i}$. From the assumption that the peak and trough are $\beta_{i}/\alpha_{i}$ and zero, respectively, the amplitude of $x_{i}$ is

(60) $$\varepsilon_{i}=\frac{\beta_{i}}{\alpha_{i}}$$

The relative change of a system property in response to a relative change in a parameter is called single-parameter sensitivity (see the following section). The single-parameter sensitivities for period are

(61) $$S_{\alpha_{i}}^{\tau }=\frac{\alpha_{i}}{\tau }\frac{\partial \tau }{\partial \alpha_{i}}=\tau^{-1}{\alpha_{i}}^{-1}\left[\text{ln}\left[\rho_{i}\left(1-\rho_{i}\right)\right]-\frac{1-2\rho_{i}}{1-\rho_{i}}\right]$$

(62) $$S_{\beta_{i}}^{\tau }=\frac{\beta_{i}}{\tau }\frac{\partial \tau }{\partial \beta_{i}}=\tau^{-1}{\alpha_{i}}^{-1}\frac{1-2\rho_{i}}{1-\rho_{i}}$$

(63) $$S_{K_{i}}^{\tau }=\frac{K_{i}}{\tau }\frac{\partial \tau }{\partial K_{i}}=-\tau^{-1}{\alpha_{i}}^{-1}\frac{1-2\rho_{i}}{1-\rho_{i}}$$

MPS is given by summing the squared single-parameter sensitivities and represents system's fragility to small, random, and simultaneous fluctuations in all kinetic parameters (see the following section). Period MPS is given by

(64) $$\begin{array}{c} {\text{Φ}}_{\tau }=\overset{n}{\underset{i=1}{\sum }}\left[{\left(S_{\alpha_{i}}^{\tau }\right)}^{2}+{\left(S_{\beta_{i}}^{\tau }\right)}^{2}+{\left(S_{K_{i}}^{\tau }\right)}^{2}\right]\\ =\frac{\overset{n}{\underset{i=1}{\sum }}\left[{\left(\frac{\text{ln}\left[\rho_{i}\left(1-\rho_{i}\right)\right]}{\alpha_{i}}-\frac{1-2\rho_{i}}{\alpha_{i}\left(1-\rho_{i}\right)}\right)}^{2}+2{\left(\frac{1-2\rho_{i}}{\alpha_{i}\left(1-\rho_{i}\right)}\right)}^{2}\right]}{{\left[\overset{n}{\underset{i=1}{\sum }}\frac{\text{ln}\left[\rho_{i}\left(1-\rho_{i}\right)\right]}{\alpha_{i}}\right]}^{2}} \end{array}$$

The single-parameter sensitivities of amplitude are

(65) $$S_{\alpha_{j}}^{\varepsilon_{i}}=\frac{\alpha_{j}}{\varepsilon_{i}}\frac{\partial \varepsilon_{i}}{\partial \alpha_{j}}=\left\{\begin{array}{c} -1\left(i=j\right)\\ 0\left(i\neq j\right) \end{array}\right)$$

(66) $$S_{\beta_{j}}^{\varepsilon_{i}}=\frac{\beta_{j}}{\varepsilon_{i}}\frac{\partial \varepsilon_{i}}{\partial \beta_{j}}=\left\{\begin{array}{c} 1\left(i=j\right)\\ 0\left(i\neq j\right) \end{array}\right)$$

(67) $$S_{K_{j}}^{\varepsilon_{i}}=\frac{K_{j}}{\varepsilon_{i}}\frac{\partial \varepsilon_{i}}{\partial K_{j}}=0$$

Amplitude MPS is given by

(68) $${\text{Φ}}_{\varepsilon_{i}}=\overset{n}{\underset{j=1}{\sum }}\left[{\left(S_{\alpha_{j}}^{\varepsilon_{i}}\right)}^{2}+{\left(S_{\beta_{j}}^{\varepsilon_{i}}\right)}^{2}+{\left(S_{K_{j}}^{\varepsilon_{i}}\right)}^{2}\right]=2$$

### Multiparameter sensitivity (MPS)

Generally a dynamic model for biochemical networks is described by ordinary differential equations:

(69) $$\overset{\cdot }{\text{x}}=\text{F}\left(t,\text{x},\text{p}\right)$$

where *t *is time, **x **is the vector whose elements are the variables for molecular concentrations, $p=\left(p_{1},p_{2},\ldots ,p_{m}\right)$ is the kinetic parameter vector, and *m *is the number of kinetic parameters. In this study, $p=\left(\alpha_{1},\ldots ,\alpha_{n},\beta_{1},\ldots ,\beta_{n},K_{1},\ldots ,K_{n}\right)$, where *n *is the number of molecular species. Let *q*(**p**) be a given system's output (oscillation period and amplitude in this study) which depends on the kinetic parameter vector. The single-parameter sensitivity of the output with respect to a change in the *i*th parameter is given by

(70) $$S_{p_{i}}^{q}=\frac{p_{i}}{q}\frac{\partial q}{\partial p_{i}}=\frac{\partial \text{ln}q}{\partial \text{ln}p_{i}}$$

Single-parameter sensitivities are used to identify parameters influential on the output.

Assuming that the relative change in the output is the linear combination of a change in each parameter, multiparameter sensitivity (MPS) [8,36-38] is given by

(71) $${\text{Φ}}_{q}=\overset{m}{\underset{i=1}{\sum }}{\left(S_{p_{i}}^{q}\right)}^{2}$$

Although MPS is expressed as the sum of the squared single-parameter sensitivities, it represents how fragile the system's output is when small, random, and simultaneous fluctuations are provided to all kinetic parameters [8]. MPS can be used as an indicator of biochemical system's robustness in a cell, where kinetic parameters constantly fluctuate. MPS is mathematically equal to the normalized variance given by the Monte-Carlo method [9-11], where all kinetic parameters are simultaneously and randomly deviated from the nominal values. For more details, see our previous work [8].

### Numerical computation of multiparameter sensitivity (MPS)

Generally, it is hard to derive the analytical solution for MPS when the given biochemical models are complicated. As a practical solution, the MPS is numerically computed by providing a small perturbation to kinetic parameters ($\alpha_{i}$, $\beta_{i}$ and $K_{i}$ in this study) [8]. Eq (70) can be rewritten as

(72) $$S_{p_{i}}^{q}=\frac{p_{i}}{q\left(p\right)}\underset{\Delta p_{i}\to 0}{\text{lim}}\frac{q\left(p^{\prime }\right)-q\left(p\right)}{\Delta p_{i}}=\underset{\Delta p_{i}\to 0}{\text{lim}}\frac{\text{ln}q\left(p^{\prime }\right)-\text{ln}q\left(p\right)}{\text{ln}\left(p_{i}+\Delta p_{i}\right)-\text{ln}p_{i}}$$

where $p=\left(p_{1},\ldots ,p_{i},\ldots ,p_{m}\right)$ is the standard parameter vector, $p^{\prime }=\left(p_{1},\ldots ,p_{i}+\Delta p_{i},\ldots ,p_{m}\right)$ is the perturbed parameter vector, and *m *is the number of kinetic parameters. In the numerical computation of single-parameter sensitivity, $\Delta p_{i}$ is not infinitesimal but a small value ($\Delta p_{i}=10^{-3}p_{i}$ in this study). The numerical computation shown here was used to obtain the numerical integration solutions and semi-analytical solutions (see Table 1). We validated the numerical computation in the previous work [8].

## Abbreviation

MPS: Multiparameter sensitivity.

## Competing interests

The authors declare that they have no competing interests.

## Authors' contributions

KM and HK participated in the design of the study. KM performed the mathematical analysis. KM and HK analyzed the data and wrote the paper. Both authors read and approved the final manuscript.

## Supplementary Material

Additional file 1 **Supplementary Figures.pdf**. Figure S1 and S2 are provided. Click here for file

## Declarations

Publication charges for this work were funded by Grant-in-Aid for Scientific Research (B) (25280107) from the Ministry of Education, Culture, Sports, Science and Technology of Japan.

This article has been published as part of *BMC systems Biology *Volume 8 Supplement 5, 2014: Proceedings of the 25th International Conference on Genome Informatics (GIW/ISCB-Asia): Systems Biology. The full contents of the supplement are available online at http://www.biomedcentral.com/bmcsystbiol/supplements/8/S5.
