# Supplementary material for: Analytical study of robustness of a negative feedback oscillator by multiparameter sensitivity
Source: BMC Syst Biol. 2014 Dec 12;8(Suppl 5):S1. doi: 10.1186/1752-0509-8-S5-S1 (PMC4305980; doi:10.1186/1752-0509-8-S5-S1)
Supplement: Additional file 1 — Supplementary Figures.pdf. Figure S1 and S2 are provided. [file 1752-0509-8-S5-S1-S1.pdf]

Additional file 1: Supplementary figures

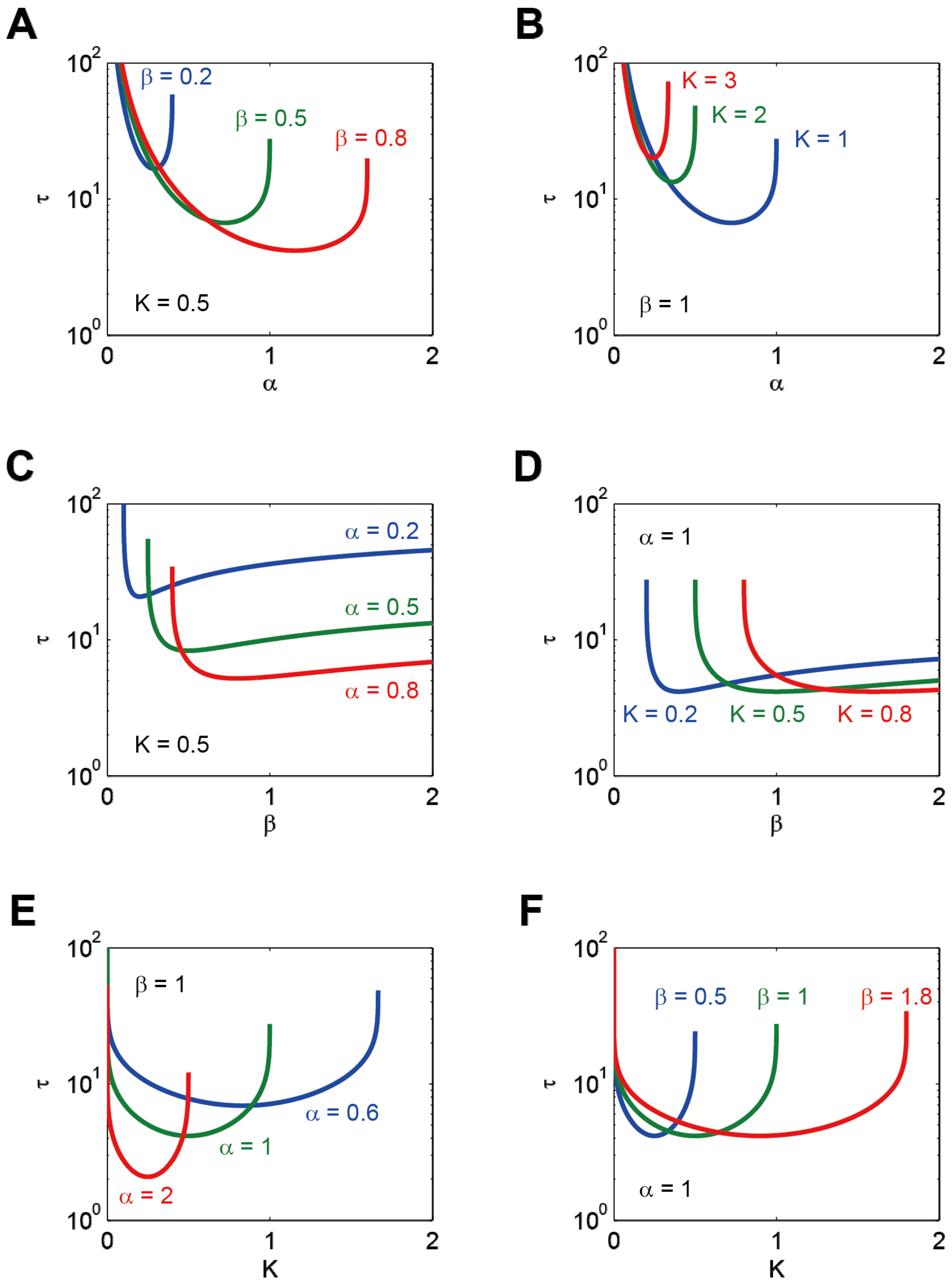

Figure S1 Effect of changes in kinetic parameters on the period

The period was calculated by Eq (9). Here, $n = 3$, $\alpha_i = \alpha$, $\beta_i = \beta$ and $K_i = K$ ($i \in \{1,2,3\}$).

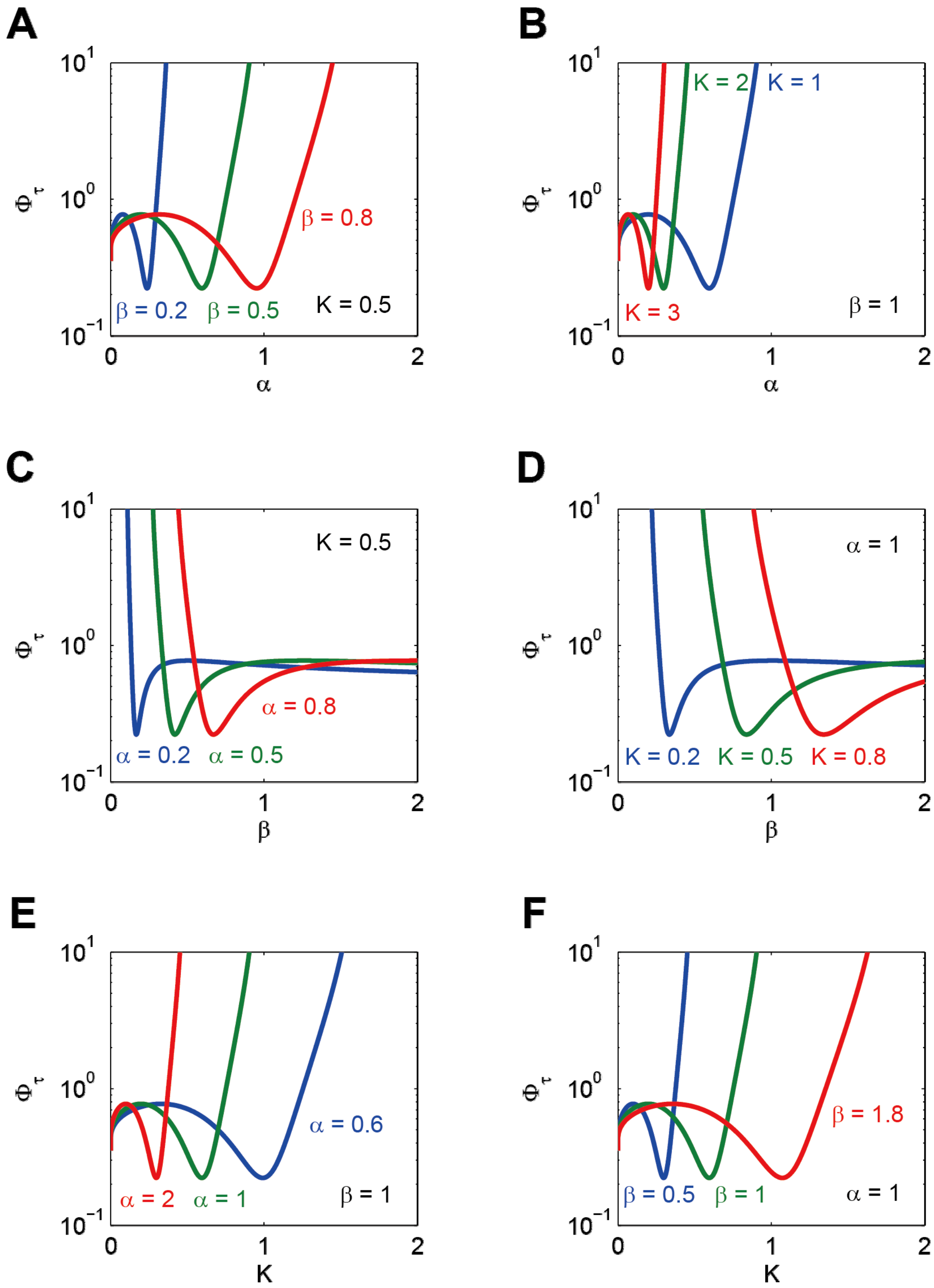

Figure S2 Effect of changes in kinetic parameters on the period MPS

The period MPS was calculated by Eq (12). Here, $n = 3$, $\alpha_i = \alpha$, $\beta_i = \beta$ and $K_i = K$ ($i \in \{1,2,3\}$).
